# Synergistic effect of exogenous multi-enzyme and phytase on growth performance, nutrients digestibility, blood metabolites, intestinal microflora and morphology in broilers fed corn-wheat-soybean meal diets

**DOI:** 10.5713/ab.20.0663

**Published:** 2021-01-01

**Authors:** MinJu Kim, Santosh Laxman Ingale, Abdolreza Hosseindoust, YoHan Choi, KwangYeol Kim, ByungJo Chae

**Affiliations:** 1 Centre for Nutrition and Food Sciences, Queensland Alliance for Agriculture and Food Innovation, The University of Queensland, QLD 4072, Australia; 2 Advanced Enzyme Technologies Ltd., Thane 400604, India; 3 College of Animal Life Sciences, Kangwon National University, Chuncheon 24341, Korea; 4 Swine Division, National Institute of Animal Science, Rural Development Administration, Cheonan 31000, Korea

**Keywords:** Exogenous Enzyme, Broiler Chickens, Phytase, Nutrients Digestibility, Gut Microflora, Growth Performance

## Abstract

**Objective:**

This study was conducted to investigate the synergistic effect of exogenous multi-enzyme and phytase on growth performance, nutrients digestibility, blood metabolites, intestinal microflora, and morphology in broilers fed corn-wheat-soybean meal diets.

**Methods:**

A 2×2 factorial design was used in this study. Four dietary treatments consisted of i) basal diets (corn-wheat-soybean meal based diets without multi-enzyme and phytase), ii) basal diets with phytase (0.05%), iii) basal diets with exogenous multi-enzyme (0.05%), and iv) basal diets with exogenous multi-enzyme including phytase (0.05%). A total of 480 broiler chickens (Ross 308 - one day old) were weighed and allotted to thirty-two cages (15 birds per cage), and chicks were randomly allocated to four dietary treatments.

**Results:**

The body weight gain and feed conversion rate were improved by supplementation of exogenous multi-enzyme containing phytase during the finisher period (p<0.05). The birds fed diets with exogenous multi-enzyme containing phytase had a significantly greater digestibility of dry matter, gross energy, crude protein, calcium, and phosphorus compared with birds fed non-supplemented diets (p<0.05). The chickens fed diets with exogenous multi-enzyme containing phytase showed a higher concentration of Ca and P in the serum (p<0.05). The population of *Lactobacillus* spp., *Escherichia coli*, and *Clostridium* were not affected in the ileum and cecum of chickens fed enzyme-supplemented diets. The dietary supplemental exogenous multi-enzyme containing phytase showed a significant improvement in villus height, crypt depth, and villus height and crypt depth ratio, compared to basal diets or dietary supplemental phytase (p<0.05).

**Conclusion:**

The supplementation of the exogenous multi-enzyme containing phytase synergistically improved the growth performance, nutrients digestibility, and villus height of the small intestine of broiler chickens fed a corn-wheat-soybean meal based diets.

## INTRODUCTION

The poultry diets are mainly comprised of the grains such as corn, wheat, and soybean meal (SBM) that contain non-starch polysaccharides (NSP) (10% to 22.7%) as anti-nutritional factors, which have physiological impacts in chickens. NSP in corn, SBM, and wheat are mainly insoluble types, as arabinoxylan and β-galactomannan in corn and α-galactosides and β-galacto-mannan in SBM, and arabinoxylan in wheat [1–4]. Insoluble NSP encapsulating nutrients inside the cell wall make them unavailable for digestion, increasing the digesta viscosity and pH, and disturbing the action of endogenous enzymes in the gut [5–8]. Feedstuff such as corn, SBM, and wheat have phytate that retain carbohydrates, microminerals, amino acids, and some digestive enzymes, which in turn impedes the absorption of nutrients in broiler diets as endogenous phytase is not produced in the gut of chickens [9].

Supplemental exogenous enzymes in broiler diets have been used to release the chemical structure of NSP and to improve the growth performance and nutrient digestibility [10–12]. Amerah et al [13] reported that increased weight gain and feed conversion ratio (FCR) was shown in boilers when a corn-SBM based diet was supplemented with exogenous multi-enzymes compared to single enzyme supplementation by decreasing ileal flow of components of soluble and insoluble non-starch polysaccharides. Furthermore, supplementation of xylanase in wheat-SBM based diet decreased ileal digesta viscosity and improved growth performance and apparent ileal digestibility of nutrients in broilers [14,15]. However, Wu et al [16] observed that supplementation of exogenous enzymes such as xylanase and phytase individually in wheat-based diet improved the growth performance and reduced the small intestinal viscosity, while the combination of phytase and xylanase caused no further improvements on broiler performance.

It is speculated that if dietary exogenous multi-enzyme and phytase are supplemented in combination in the diets, a synergistic effect would be shown in broiler chickens. This study was conducted to investigate the effect of exogenous multi-enzyme including phytase on growth performance, nutrients digestibility, blood metabolites, gut microbiota, and small intestinal morphology in broilers fed corn-wheat-SBM diets.

## MATERIALS AND METHODS

The experiment was conducted at the facility of Kangwon National University farm and all animal experimental procedures and protocol in this research were approved by the Institutional Animal Care and Use Committee of Kangwon National University, Republic of Korea (Approval No: KW-141111-1).

### Animals, diets, and experimental design

A randomized complete block design consisting of a 2×2 factorial design was used in this study. The enzymes used in this experiment were supplied by Advanced Enzyme Technologies Ltd. (Thane, India). Exogenous multi-enzymes used in this study were two types, enzyme blend as amylase, protease, mannanase, and xylanase and enzyme blend including phytase. The treatments included: i) basal diets (corn-wheat-SBM based diets without enzyme), ii) basal diets with phytase (DigePhos 5G, Advanced Enzyme Technologies, India) (0.05%), iii) basal diets with exogenous multi-enzyme (DigeGrain Delta, Advanced Enzyme Technologies, India) (0.05%), and iv) basal diets with exogenous multi-enzyme including phytase (0.05%). A total of 480 one-day old Ross 308 chicks (47.74±0.8 g, body weight [BW]) were randomly allotted to 8 replicates of 15 birds per treatment. This experiment was conducted for a total of 35 days (phase 1, d 0 to 21 and phase 2, d 22 to 35). The nutrient contents of experimental diets (Table 1) were formulated in order to meet or exceed nutrient requirements by Nutrient Requirements of Poultry [17]. The birds were raised in floor pens with rice husks used as beddings (about 5 cm in depth). Each pen was equipped with a hopper feeder and five water nipple drinkers connected to water tank, allowing the birds to take feed and water “*ad libitum*”. The chicks were kept at a temperature of 34°C for d 1 to 3, and thereafter the temperature was gradually decreased to 23°C according to normal management practices. All birds were under a continuous lighting for 23 h/d.

### Experimental procedure and sampling

The BW and feed intake (FI) of chickens per pen were measured at the start and end of each phase for calculation of the body weight gain (BWG) and FCR. To determine the apparent total tract digestibility (ATTD), chromic oxide (Cr, 0.25%) was added in each diet as a marker. Two birds from each pen were allocated in individual cages and fed experimental diets for 10 days, 5 d for adaptive period and 5 d for sampling period. The excreta samples from each pen were pooled into stainless trays to analyze ATTD of dry matter (DM), gross energy (GE), crude protein (CP), calcium (Ca), and phosphorus (P). The pooled excreta samples per treatment were dried in a forced-air oven at 60°C for 72 h. Thereafter, the dried samples were ground by an electric feed grinder (Thomas Model 4 Wiley Mill, Thomas Scientific, Swedesboro, NJ, USA) and sieved through a 1-mm screen for chemical analysis.

Two birds per pen were randomly chosen for blood collection. Blood samples were taken from the wing vein with disposable vacutainers containing heparin sodium at d 21 and d 35 and centrifuged at 3,000 rpm for 15 min at 4°C. Then, the supernatant of samples was moved to 1 mL tube by pipette and stored at −20°C before analysis of blood metabolites. Three birds per replicate were slaughtered at d 35 to collect samples for analysis of the apparent ileal digestibility (AID) of nutrients, gut microflora in ileum and cecum, and small intestinal morphology. The ileum and cecum contents were stored in sterilized plastic bottles at 7°C and transferred to laboratory immediately for bacterial analysis. The intestinal samples (approximately 3 cm in length) were acquired from the duodenum (middle portion of the duodenal loop), jejunum (midway between the distal portion of the duodenal loop and Meckel’s diverticulum), and ileum (midway between Meckel’s diverticulum and ileocecal junction). The intestinal contents were washed with phosphate-buffered saline and the samples were fixed by buffered formalin (10%) for analysis of the intestinal morphology.

### Chemical and microbial analysis

Experimental diets and excreta samples were analyzed according to methods described by AOAC [18] in triplicate for DM (930.15), CP (990.03), and ash (942.05) Ca, and P (985.01). The GE was assessed by using a bomb calorimeter (Model 1261, Parr Instrument Co., Moline, IL, USA), and chromium (Cr) concentration was measured with an automated spectrophotometer (Jasco V-650, Jasco Corp., Tokyo, Japan) according to the procedure of Fenton and Fenton [19]. Analysis of serum metabolites was conducted by using an automated chemistry analyzer (Fuji Drichem 3500i; Fujifilm Corp., Tokyo, Japan).

The microbiological assay was carried out by the procedure suggested by Kim et al [20]. The excreta samples from ileum and cecum per replicate were pooled in a sterilized plastic bottles and mixed. The samples (1 g) were put together in anaerobic dilution solution (9 mL) by keeping it under CO_2_. The serial dilutions (10^−^^5^ to 10^−^^8^) were made by using the anaerobic dilution solution, and 1 mL diluted solution was plated onto agar plates in triplicate. The total anaerobic bacteria were determined on plate count agar (TAB, Difco Laboratories, Detroit, MI, USA) and incubated at 37°C for 24 h under anaerobic condition. The *Lactobacillus* spp. was determined on MRS agar (with 0.20 g/L NaN_3_+0.50 g/L L-cystine hydrochloride monohydrate) and incubated at 39°C for 48 h under aerobic conditions. The *Clostridium* spp. was determined on tryptose sulphite cycloserine agar (Oxoid, Hampshire, UK) and incubated at 37°C for 24 h under anaerobic conditions. The *Escherichia coli* (*E. coli*) was determined on MacConkey agar and incubated at 37°C for 24 h under aerobic conditions. The anaerobic condition was made in a gaspack anaerobic system (BBL, No. 260678, Difco, USA).

### Small intestinal morphology

The samples of duodenum, jejunum and ileum were processed with ethanol for one day and were embedded in standard paraffin. Intestinal samples were stained with azure A and eosin and then well-oriented cryptvillus groups (total 10 intact) were selected for intestinal cross-sections. The samples were measured in triplicates by using an optical microscope and image analysis system (Media Cyber genetics, Optimus software version 6.5, North Reading, MA, USA). Villus height (VH, μm) was determined from the top of the villus to crypts of Lieberkühn and crypt depth (CD, μm) was measured as the depth of the invagination between adjacent villi. All morphological characteristics were analyzed in 10-μm increments.

### Statistical analyses

All data was analyzed by general linear model procedure of SAS (SAS Inst. Inc., Cary, NC, USA) as 2×2 factorial arrangement with dietary treatments of exogenous multi-enzyme and phytase as main effects. Significant differences among the treatment means were partitioned by Tukey’s honestly significant difference test at p<0.05 statistical level, and 0.05< p<0.10 was considered a trend towards significance. Pens were considered the experimental unit for growth performance and broiler chickens were considered the experimental unit for measuring the nutrients digestibility, blood metabolites, gut microbiota, and intestinal morphology.

## RESULTS

### Growth performance

The results of growth performance are presented in Table 2. The broilers fed diet supplemented with multi-enzyme and phytase improved significantly in the BWG and FCR at starter and finisher phases (p<0.05). Furthermore, the supplementation of multi-enzyme and phytase in combination had an interaction effect in BWG during finisher phase (p<0.05). The broilers fed diet supplemented with multi-enzyme with phytase had significantly better FCR than broilers fed diet supplemented with multi-enzyme or phytase individually (p<0.05).

### Digestibility of nutrients

The results of nutrients digestibility in feces and ileum are shown in Table 3. The exogenous multi-enzyme significantly improved the ATTD and AID of DM, GE, and CP (p<0.05) and phytase enhanced the digestibility of Ca and P in both ATTD and AID (p<0.05). No interaction between exogenous multi-enzyme and phytase was observed. The exogenous multi-enzyme containing phytase supplementation showed the significant increases of ATTD and AID in DM, GE, CP, Ca, and P compared to basal diets, supplemental phytase without multi-enzyme, or supplemental multi-enzyme without phytase treatment (p<0.05).

### Blood metabolites

The results of blood metabolites are shown in Table 4. There was no interaction between exogenous multi-enzyme and phytase. The concentration of Ca and P in serum was significantly increased with phytase supplementation on d 21 and d 35 (p<0.05). The treatment supplemented with exogenous multi-enzyme containing phytase had the highest concentration of Ca and P in serum amongst the four treatment groups (p<0.05).

### Ileum and cecum microflora

The results of intestinal microflora are shown in Table 5. No significant effects on multi-enzyme, phytase and their interaction were observed (p>0.05). There were no difference in population of *Lactobacillus* spp., *E. coli*, and *Clostridium* spp. in the ileum and cecum of chicken fed diet supplemented with exogenous multi-enzyme.

### Small intestinal morphology

The results of intestinal morphology are presented in Table 6. The exogenous multi-enzyme with phytase significantly increased the VH and decreased the CD in all intestinal segments (p<0.05). However, no interaction in supplementation of exogenous multi-enzyme and phytase was observed. The exogenous multi-enzyme containing phytase showed the most significant increase of VH, decrease of CD, and improvement of villus height and crypt depth ratio (VH:CD) comparing to basal diets or dietary supplemental phytase treatment group (p<0.05).

## DISCUSSION

In this study, dietary supplementation of phytase and multi-enzyme in combination improved the BWG and feed efficiency in broilers fed corn-wheat-SBM based diets. The supplementation of exogenous enzymes can improve nutrient utilization in the cereal grains by decreasing the intestinal viscosity in broilers [21]. The growth performance was in agreement with results reported by Singh et al [22], who observed that supplementation of the combined enzymes such as xylanase, amylase, protease, and phytase increased the BWG by about 10% to 15% and decreased FCR by 0.06 to 0.11 units in broilers fed corn-SBM diet or corn-wheat-SBM based diet. However, Yaghobfar and Kalantar [23] reported that supplementation of glycanase and phytase in combination in corn-wheat based diet did not affect the weight gain and feed conversion rate of broilers. The different effects of carbohydrase and phytase on growth performance of broilers would be due to feed ingredients and enzyme type [24]. Wheat is rich source of arabinoxylans that consists of a backbone of β-(1,4)-linked xylose residues, which are substituted with arabinose residues on the C(O)-2 and/or C(O)-3 position [25]. The supplemental xylanase and phytase are required in poultry diets based on wheat. Xylanase degrades arabinoxylans in the cell wall, releasing encapsulated starch and other nutrients from inside the cell wall, at the same time as reducing digesta viscosity caused by the soluble fraction [26]. Furthermore, phytase hydrolyze phosphate from phytic acid in a sequential process, resulting a myoinositol and phosphate with the liberated nutrients, which were linked to phytic acid [27,28]. The combination of glycosidase and phytase can increase the accessibility to phytate more than being used individually [21]. That phytate molecules in wheat encased proteins in the aleuron layer by complex carbohydrates [29]. In this study, exogenous multi-enzyme and phytase synergistically improved not only ATTD but also AID in DM, GE, CP, Ca, and P. The improvement in nutrients digestibility may be associated with dietary enzymes including amylase, protease, β-mannanase, and xylanase, and phytase to degrade dietary NSP. In agreement, Lu et al [24] obtained that the use of xylanase, β-glucanase, and phytase in combination improved the DM, GE, CP, and P in chickens fed the ME (Please write it in full form.), CP, Ca, and P deficient diet during the growth phase. Furthermore, the improved protein digestibility was obtained when xylanase and β-glucanase were supplied in wheat-based diets [30]. However, Cowieson and Adeola [31] showed no interactive effects of xylanase, amylase, protease and phytase on nutrients digestibility and ileal digestible energy. Dietary supplemental β-mannanase that hydrolyzes β-mannan to the 1,4-β-D mannan chain of glucomannans and galactomannans can ameliorate energy and nutrient utilization in SBM as containing β-mannan, β-glucomannan, and β-galactomannan [32,33]. Li et al [34] reported that supplementation of β-mannanase improved the apparent metabolizable energy and protein digestibility of broilers. In addition, it was reported that the multi-enzyme containing xylanase, amylase, protease, and phytase in ME and P deficient diets showed a synergistic increase on growth performance and ileal phosphorus digestibility [35]. The Ca and P concentration in serum were increased with supplementation of exogenous multi-enzyme and phytase in combination with an increase of digestibility on this study. A similar result was observed by Lu et al [24] who reported that the addition of xylanase, β-glucanase, and phytase in combination in deficient diets increased the P concentration in serum of broiler chickens.

The gastrointestinal microbiota is one of the most factors determining immunity and physiological systems of broiler chickens. Pathogenic bacteria such as coliforms enhance infections and decrease the growth performance of chickens [36,37]. In this study, the supplementation of multi-enzyme showed a tendency for increasing *Lactobacillus* population and decreasing the colonization of *E. coli* and *Clostridiums* spp. in the ileum and cecum. The effect of exogenous enzyme such as β-mannanase and xylanase on intestinal microorganism have been observed in previous studies [36–39]. Roofchaei et al [40] detected that the dietary supplemental xylanase, β-glucanase, and phytase in combination had an inhibitory effect on the *E. coli* population in the ileum. It is well described that the oligosaccharides caused from action of xylanase or β-mannanase may alter the microbial population in the intestinal tract of broilers [41,42]. Xylanase degrades arabinoxylan, a major NSP found in wheat, producing shorter chain xylo-oligomers that are involved in production of short chain fatty acid (SCFA) in hindgut of broilers [43]. The produced SCFA may instigate environmental conditions favorable for lactic acid bacteria such as *Lactobacillus* spp. [44], which impedes pathogenic microbial propagation [45]. Furthermore, Yaghobfar and Kalantar [23] reported that the decrease of *E. coli* population and increase of *Lactobacillus* population in the ileum were observed by supplementation of β-glucanase and phytase. Phytase can alter the digesta pH and the ileal microbial population by reducing the buffering capacity of a diet [46,47].

In the present study, the broilers fed a diet supplemented with the combination of multi-enzyme and phytase improved villus height and crypt depth in duodenum, jejunum, and ileum. Dietary β-mannan (derived from SBM) and arabinoxylans (derived from wheat) cause an increase of digesta viscosity, which provokes change in the intestinal mucosa by accessing the mucosal surface to the viscous ingredients [42,48]. The digesta viscosity leads to quick changes in the intestinal mucosa due to the proximity of the mucosal surface to the intestinal viscose materials [23]. Therefore, grains such as wheat and SBM may induce shorter VH or deeper CD by stimulating division and tissue renewal of intestinal cells [49]. The supplementation of xylanase and β-mannanase in corn-wheat-SBM based diets might have alleviated the increase of digesta viscosity of broilers in this study. In a study [50], dietary supplemental xylanase decreased the digesta viscosity of in the jejunum of broilers. Furthermore, the effect on the small intestinal morphology might be due to butyrate production in hindgut. The supplementation of xylanase and β-mannanase in diet containing wheat and SBM lead to release xylo-oligosaccharides and manno-oligosaccharides, respectively in gut of broilers [42,45,51], producing butyrate that plays a role as stimulator of glucagon-like peptide 2 (GLP-2) production. This hormone is secreted by entero-endocrine L cells localized in stomach, small intestine, colon and subepithelial myofibroblasts and acts as multiple downstream mediators [52]. Hu et al [53] reported that GLP-2 injection increased the small intestinal weight and the villus height of duodenum and jejunum of broiler chickens. The results in this study are in accordance with the observations of Roofchaei et al [40] who reported that dietary supplemental xylanase, β-glucanase, and phytase in combination increased villus length compared to control diet without supplemental enzyme.

In conclusion, the addition of phytase and multi-enzyme in broiler diet consisted of corn, wheat, and SBM showed synergistical improvement on growth performance and utilization of nutrients. Furthermore, the increased intestinal villus would be more likely from synergistical effect of supplementation with multi-enzyme containing xylanase and β-mannanase, and phytase. Our study suggests that supplemental xylanase, β-mannannase, and phytase in corn-wheat-SBM based diets may derive advantages in broiler chickens.

## Figures and Tables

**Table 1:** Formula and chemical composition of experimental diets (as-fed basis)

Items	Starter (d 0–21)	Finisher (d 21–35)
Ingredient (%)		
Corn	53.47	50.92
Wheat	5.00	10.00
Soybean meal (45%)	27.98	23.10
Fish meal (60%)	2.00	-
Corn gluten	4.36	7.00
Soy-oil	2.77	4.47
Threonine (98.5%)	0.07	0.07
Lysine (78%)	0.17	0.25
Methionine (50%)	0.56	0.43
Tryptophan (10%)	-	0.02
Choline	0.05	0.05
Limestone	1.76	1.75
MDCP	1.11	1.24
Salt	0.20	0.20
Mineral premix^1)^	0.20	0.20
Vitamin premix^2)^	0.30	0.30
Total	100.00	100.00
Nutrient composition (%)		
Metabolic energy (Kcal/kg)	3,050	3,150
Crude protein	22.00	20.00
Crude protein (analyzed)	22.13	20.08
Crude fiber (analyzed)	2.62	2.51
Crude ash (analyzed)	2.65	2.44
Ether extract (analyzed)	4.72	4.50
Calcium	1.00	0.90
Available phosphorus	0.40	0.38
Lysine	1.20	1.07
TSAA	0.94	0.80
Threonine	0.80	0.70
Tryptophan	0.22	0.19

MDCP, mono-dicalcium phosphate; TSAA, total sulfur amino acids.

1) Supplied per kg diet: 20 mg Fe, 16 mg Cu, 110 mg Zn, 120 mg Mn, 1.25 mg I, 0.9 mg Co, 0.3 mg Se.

2) Supplied per kg diet: 10,000 IU vitamin A, 4,500 IU vitamin D_3_, 65 mg vitamin E, 1.5 mg vitamin B_1_, 12 mg vitamin B_2_, 3.2 mg vitamin B_6_, 0.011 mg vitamin B_12_, 3.0 mg vitamin K_3_, 18 mg pantothenic acid, 60 mg niacin, 0.18 mg biotin, 1.9 mg folic acid, 18 mg ethoxyquin.

**Table 2:** Effects of dietary supplementation of exogenous multi-enzyme containing phytase on growth performance in broilers

Items	−Enzyme		+Enzyme^1)^		SEM	p-value		
		
	−Phytase	+Phytase	−Phytase	+Phytase		E	P	E×P
Starter (d 0 to 21)								
BWG (g)	774^b^	807^a^	812^a^	827^a^	7.16	<0.001	0.003	0.241
FI (g)	1,176	1,186	1,185	1,190	11.68	0.603	0.526	0.817
FCR	1.52^a^	1.47^ab^	1.46^b^	1.44^b^	0.02	0.016	0.063	0.454
Finisher (d 21 to 35)								
BWG (g)	1,096^c^	1,136^b^	1,143^b^	1,160^a^	3.47	<0.001	<0.001	0.003
FI (g)	2,067	2,113	2,103	2,054	19.78	0.589	0.942	0.029
FCR	1.89^a^	1.86^a^	1.84^a^	1.77^b^	0.02	0.001	0.012	0.234
Overall (d 0 to 35)								
BWG (g)	1,867^c^	1,943^b^	1,955^b^	1,988^a^	7.98	<0.001	<0.001	0.018
FI (g)	3,243	3,299	3,288	3,244	23.60	0.832	0.803	0.044
FCR	1.74^a^	1.70^b^	1.68^b^	1.63^c^	0.01	<0.001	0.001	0.724

E, multienzyme; P, phytase; SEM, standard error of means; BWG, body weight gain; FI, feed intake; FCR, feed conversion ratio.

1) Enzyme, multi-exogenous enzyme contained with amylase, protease, mannanase, and xylanase.

a,b,c Means within a row with unlike superscripts differ significantly (p<0.05).

**Table 3:** Effects of dietary supplementation of exogenous multi-enzyme containing phytase on ATTD and AID (%) in broilers

Items	−Enzyme		+Enzyme^1)^		SEM	p-value		
		
	−Phytase	+Phytase	−Phytase	+Phytase		E	P	E×P
ATTD								
DM	74.05^b^	74.52^b^	74.86^ab^	75.55^a^	0.33	0.011	0.097	0.733
GE	75.05^b^	75.67^ab^	75.88^ab^	76.52^a^	0.29	0.016	0.064	0.954
CP	66.51^c^	66.92^bc^	68.20^ab^	68.62^a^	0.54	0.004	0.457	0.995
Ca	40.03^b^	41.84^ab^	40.71^ab^	42.31^a^	0.64	0.380	0.014	0.875
P	38.39^c^	40.38^ab^	39.26^abc^	41.03^a^	0.66	0.265	0.009	0.865
AID								
DM	75.47^b^	76.03^ab^	76.29^ab^	77.02^a^	0.34	0.018	0.083	0.812
GE	76.52^b^	77.09^b^	77.41^ab^	78.35^a^	0.37	0.015	0.079	0.660
CP	67.93^b^	68.35^b^	69.46^ab^	70.09^a^	0.54	0.006	0.343	0.849
Ca	41.30^b^	43.02^ab^	41.90^ab^	43.78^a^	0.67	0.330	0.013	0.908
P	39.98^b^	41.85^ab^	40.67^ab^	42.69^a^	0.68	0.271	0.008	0.913

E, multienzyme; P, phytase; SEM, standard error of means; ATTD, apparent total tract digestibility; AID, apparent ileal digestibility; DM, dry matter; GE, gross energy; CP, crude protein; Ca, calcium; P, phosphorus.

1) Enzyme, multi-exogenous enzyme contained with amylase, protease, mannanase, and xylanase.

a,b,c Means within a row with unlike superscripts differ significantly (p<0.05).

**Table 4:** Effects of dietary supplementation of exogenous multi-enzyme containing phytase on blood metabolites in broilers

Items	−Enzyme		+Enzyme^1)^		SEM	p-value		
		
	−Phytase	+Phytase	−Phytase	+Phytase		E	P	E×P
Glucose (mg/dL)								
21 d	230.50	226.38	228.38	221.50	3.33	0.314	0.119	0.690
35 d	254.38	240.00	251.50	237.75	6.29	0.687	0.034	0.961
Triglyceride (mg/dL)								
21 d	31.25	28.25	24.63	28.38	2.10	0.144	0.863	0.129
35 d	25.13	27.50	27.13	26.63	0.88	0.542	0.312	0.126
Cholesterol (mg/dL)								
21 d	130.13	128.25	125.25	125.50	2.31	0.112	0.729	0.651
35 d	122.00	126.38	121.13	124.00	2.09	0.446	0.095	0.724
Ca (mg/dL)								
21 d	10.19^b^	10.54^ab^	10.35^ab^	10.68^a^	0.12	0.254	0.014	0.923
35 d	10.26^b^	10.68^ab^	10.45^ab^	10.85^a^	0.16	0.279	0.020	0.970
P (mg/dL)								
21 d	7.29^b^	7.70^ab^	7.59^ab^	7.93^a^	0.15	0.087	0.017	0.802
35 d	7.51^b^	8.01^ab^	7.78^ab^	8.23^a^	0.17	0.186	0.011	0.888

SEM, standard error of means; E, multienzyme; P, phytase; Ca, calcium; P, phosphorus.

1) Enzyme, multi-exogenous enzyme contained with amylase, protease, mannanase, and xylanase.

a,b Means within a row with unlike superscripts differ significantly (p<0.05).

**Table 5:** Effects of dietary supplementation of exogenous multi-enzyme containing phytase on ileum and cecum microflora in broilers

Items	−Enzyme		+Enzyme^1)^		SEM	p-value		
		
	−Phytase	+Phytase	−Phytase	+Phytase		E	P	E×P
Total anaerobic bacteria								
Ileum	10.08	9.91	9.92	9.91	0.06	0.204	0.166	0.208
Cecum	10.11	9.95	9.96	9.96	0.06	0.219	0.204	0.197
*Lactobacillus* spp.								
Ileum	8.89	9.25	9.27	9.31	0.12	0.073	0.101	0.195
Cecum	9.15	9.51	9.54	9.60	0.12	0.061	0.098	0.241
*Escherichia coli*								
Ileum	7.53	7.28	7.26	7.23	0.08	0.081	0.111	0.211
Cecum	7.66	7.40	7.37	7.33	0.09	0.069	0.129	0.255
*Clostridium* spp.								
Ileum	7.36	7.04	7.08	7.03	0.10	0.175	0.092	0.197
Cecum	7.58	7.30	7.23	7.25	0.11	0.081	0.261	0.191

SEM, standard error of means; E, multienzyme; P, phytase.

1) Enzyme, multi-exogenous enzyme contained with amylase, protease, mannanase, and xylanase.

**Table 6:** Effects of dietary supplementation of exogenous multi-enzyme containing phytase on intestinal morphology in broilers

Items	−Enzyme		+Enzyme^1)^		SEM	p-value		
		
	−Phytase	+Phytase	−Phytase	+Phytase		E	P	E×P
Duodenum								
VH (μm)	1,691.10^b^	1,703.52^b^	1,722.42^ab^	1,742.72^a^	12.86	0.011	0.216	0.762
CD (μm)	160.95^a^	155.71^ab^	139.84^ab^	132.67^b^	8.76	0.018	0.485	0.913
VH/CD	10.79^b^	11.18^ab^	12.73^ab^	13.57^a^	0.83	0.015	0.466	0.791
Jejunum								
VH (μm)	854.88^b^	879.27^b^	917.33^ab^	958.62^a^	25.42	0.010	0.208	0.743
CD (μm)	121.79^a^	108.44^ab^	98.09^ab^	90.91^b^	9.76	0.047	0.310	0.758
VH/CD	7.67^b^	8.85^ab^	9.78^ab^	11.65^a^	1.14	0.043	0.201	0.772
Ileum								
VH (μm)	702.91^b^	714.83^b^	731.90^ab^	753.04^a^	12.62	0.013	0.203	0.719
CD (μm)	115.90^a^	102.97^ab^	93.73^ab^	90.26^b^	7.92	0.043	0.327	0.570
VH/CD	6.34^b^	7.16^ab^	8.95^a^	8.67^ab^	0.81	0.026	0.756	0.535

SEM, standard error of means; E, multienzyme; P, phytase; VH, villus height; CD, crypt depth.

1) Enzyme, multi-exogenous enzyme contained with amylase, protease, mannanase, and xylanase.

a,b Means within a row with unlike superscripts differ significantly (p<0.05).
